# A two-neuron system for adaptive goal-directed decision-making in *Lymnaea*

**DOI:** 10.1038/ncomms11793

**Published:** 2016-06-03

**Authors:** Michael Crossley, Kevin Staras, György Kemenes

**Affiliations:** 1 Sussex Neuroscience, School of Life Sciences, University of Sussex, 1 Lewes Road, Brighton BN1 9QG, UK

## Abstract

During goal-directed decision-making, animals must integrate information from the external environment and their internal state to maximize resource localization while minimizing energy expenditure. How this complex problem is solved by the nervous system remains poorly understood. Here, using a combined behavioural and neurophysiological approach, we demonstrate that the mollusc *Lymnaea* performs a sophisticated form of decision-making during food-searching behaviour, using a core system consisting of just two neuron types. The first reports the presence of food and the second encodes motivational state acting as a gain controller for adaptive behaviour in the absence of food. Using an *in vitro* analogue of the decision-making process, we show that the system employs an energy management strategy, switching between a low- and high-use mode depending on the outcome of the decision. Our study reveals a parsimonious mechanism that drives a complex decision-making process via regulation of levels of tonic inhibition and phasic excitation.

## Abstract (web summary)

Integrating information from both the external environment and an organism's internal state is an important aspect of feeding-related decision making. Here, the authors identify a two neuron circuit within the mollusc *Lymnaea* that adapts feeding behaviour according to food availability and motivational state.

## Graphical abstract

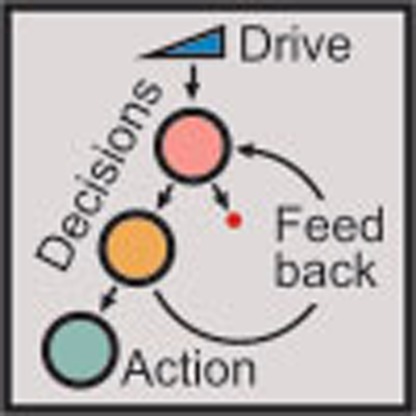

All animals face the challenge of making adaptive decisions in response to sensory stimuli encountered in their environment. Perceptual decision-making represents the simplest category where a choice is made between two actions based on the presence of one or more inputs^1^. More complex computations are required for decisions made during goal-directed behaviours^2^. Here an animal actively searches for a particular stimulus, usually driven by a homeostatic motivational state (for example, thirst and hunger). Once it makes a perceptual decision about the presence of the stimulus, it often needs to perform further adaptive decisions to maximize the chances of achieving the goal, for example, changing search strategy if insufficient resource is localized. In the face of limited resources, an important additional demand is that the goal is achieved with minimal energy expenditure. How these multiple challenges are met by the nervous system is poorly understood.

Here using both whole-animal behaviour and circuit analysis in reduced preparations, we elucidate the neural mechanisms of motivational-state-dependent decision-making during food-searching behaviour in the mollusc *Lymnaea*, an established system to study both motivational state^3^ and decision-making^4^ at single-cell resolution. Food searching is an example of a goal-directed behaviour that is essential for survival. In this case, an animal must be able to accurately judge the presence or absence of potential food and make adaptive decisions to maximize food intake with minimal energetic cost. *Lymnaea* searches for food by actively sampling its environment. This exploratory behaviour is manifested as appetitive bites performed with the mouthparts, which resemble the consummatory bites occurring during feeding and are generated by the same central pattern generator (CPG) and motoneuron networks^5^. Although the *Lymnaea* feeding circuitry has been characterized extensively^6^,^7^,^8^,^9^,^10^,^11^ (see ref. 12 for review), the neuronal mechanisms responsible for allowing the animal to detect the presence of food during food-searching behaviour and switch from appetitive to consummatory biting are not known. Likewise, although a shifting balance of reciprocal inhibition between a CPG neuron and a multifunctional modulatory interneuron was previously shown to underlie satiety-state-related differences in the frequency of spontaneous appetitive bites^3^, how this influences food-searching behaviour on a cycle-by-cycle basis remains unclear. Finally, there is a substantial knowledge deficit in explaining how interactions between the ‘motivation-encoding circuitry' and a potentially independent ‘food-detecting circuitry' lead to the decision to continue versus terminate appetitive biting. Clarification of the above mechanisms would validate *Lymnaea* as an important model system for explaining how a small defined circuit can achieve the complex task of goal-directed decision-making.

We demonstrate that the core decision-making system consists of just two neuron types—a phasically firing command-like excitatory neuron, encoding the presence of food, and a tonically firing modulatory neuron, acting as a gain controller for the animal's motivational state—and we characterize these two pathways and their interactions at the point that a decision is made. Moreover, we show that the system uses an energy-saving strategy, whereby non-essential components of the feeding motor program are attenuated. Our results therefore provide a detailed cellular understanding of the role of regulating levels of state-dependent tonic inhibition and stimulus-dependent phasic excitation during an energy-efficient decision-making process and could inform new work aimed at the identification of the core neuronal mechanisms underlying similar decision-making processes in other model systems.

## Results

### Motivational state influences food-searching decisions

**Figure 1:**
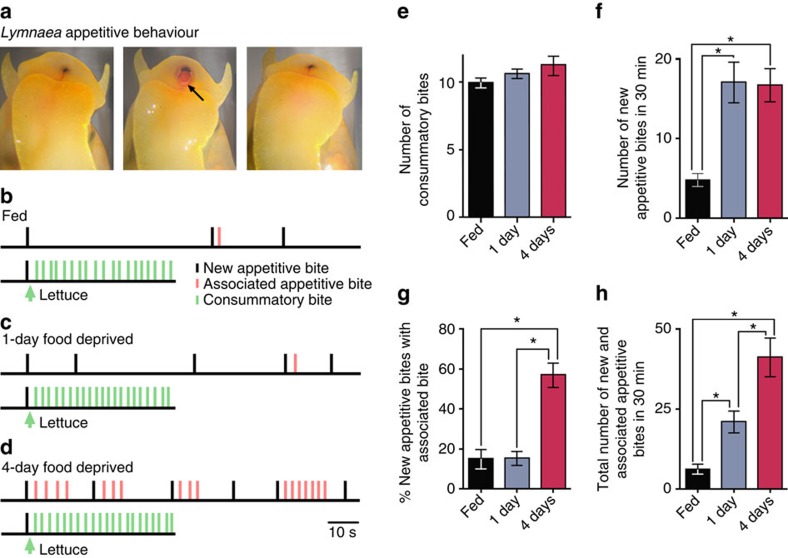
Decision-making in *Lymnaea* during food-searching behaviour. (**a**) Images of *Lymnaea*'s food-searching behaviour. (Frame 1) Animal at the water surface. (Frame 2) Appetitive bite behaviour with mouth opening and protraction of toothed radula (arrowed). (Frame 3) Retraction of radula. The same basic motor pattern is used for ingestion in the presence of food, for example, lettuce. (**b**–**d**) Example traces of *Lymnaea*'s biting behaviour in the presence and absence of food at three different levels of satiety: fed, 1-day and 4-day food deprived. Each vertical line represents a single bite. In the bottom trace of each panel, lettuce was presented at the onset of a new appetitive bite. (**e**) Histogram showing the number (mean±s.e.m.) of consummatory bites in 30 s after the appetitive bite in the presence of lettuce for each motivational state (one-way ANOVA, *P*>0.05, *n*=14). (**f**) Histogram showing the number (mean±s.e.m.) of new appetitive bites during a 30-min observation period in the absence of lettuce. One-way ANOVA, *P*<0.0001. Asterisks indicate significance confirmed by Tukey's tests (fed versus 1-day food deprived and fed versus 4-day food deprived, *P*<0.001). Tukey's tests for 1-day food deprived versus 4-day food deprived, *P*>0.05. (**g**) Histogram showing the percentage (mean±s.e.m.) of appetitive bites that had an associated bite in the absence of lettuce. One-way ANOVA, *P*<0.0001. Tukey's tests, 4-day food deprived versus fed and 4-day food deprived versus 1-day food deprived, *P*<0.001, fed versus 1-day food deprived, *P*>0.05. (**h**) Histogram showing the total number (mean±s.e.m.) of food-searching bites (new appetitive and associated appetitive bites) in the absence of lettuce. One-way ANOVA, *P*<0.0001. Tukey's tests, fed versus 1-day food deprived, *P*<0.05; fed versus 4-day food deprived, *P*<0.001; 1-day food deprived versus 4-day food deprived, *P*<0.01.

*Lymnaea* exhibits two modes of feeding behaviour; an appetitive one where the local environment is actively sampled (Fig. 1a; Fig. 1b–d, top traces) and a consummatory one in the presence of food (Fig. 1b–d, bottom traces). The basis for the judgment about the presence or absence of food and the choice between the two behavioural modes offers a highly suitable task to study the neuronal mechanisms of decision-making in a goal-directed behaviour.

To characterize the behavioural features of *Lymnaea*'s food-searching decisions, we observed animals in the presence and absence of food during appetitive feeding behaviour. Specifically, we focused on whether an appetitive bite was followed by an associated bite, as a read-out of the animal's judgment regarding the presence or absence of food. Before testing, animals were allowed to feed *ad libitum* (fed animals). Despite this, when presented with food during a new appetitive bite (defined as a bite that occurred >6 s after a previous appetitive bite, for rationale of this time window see Methods), 100% of animals tested (*n*=14) performed an associated bite (defined as a bite that occurred ≤6 s after a previous appetitive bite) and entered into a period of highly organized consummatory bites (Fig. 1b, bottom trace; Fig. 1e). In the absence of food, however, only a small proportion of the infrequent new appetitive bites produced by fed animals (Fig. 1b, top trace; Fig. 1f) had an associated bite (15±3.5%, Fig. 1g). These findings suggest that if no food is encountered during an occasional appetitive bite, a judgment is made and the animal re-enters the quiescent state. Taken together, these results indicate that *Lymnaea* have a simple mechanism to judge the presence or absence of food during food-searching behaviour and perform an appropriate response.

An important factor in decision-making during goal-directed behaviours is the animal's motivational state. Next, we considered how this impacted on the decision-making outcome using animals that were food deprived for either 1 or 4 days before testing; protocols that should serve to increase their motivational drive to locate food. Similar to fed animals, all animals from the food-deprived groups responded to food presented during an appetitive bite by performing an associated bite and switching into a bout of consummatory behaviour (Fig. 1c,d, bottom traces; Fig. 1e). There was also no difference in the interbite interval of the consummatory bites between the motivational states (fed, 3.2±0.2 s, *n*=14; 1-day food deprived, 2.8±0.1 s, *n*=14; 4-day food deprived, 2.9±0.2 s, *n*=14; one-way analysis of variance (ANOVA), *P*>0.05). Therefore, the mechanisms underlying the judgment of presence of food during the appetitive bite are not affected by motivational state. However, in the absence of food during the appetitive bite, *Lymnaea* showed marked changes in their subsequent feeding behaviour with different motivational states (Fig. 1b–d, top traces). First, there was a significant increase in the number of new appetitive bites in both food-deprived groups compared with the fed group (Fig. 1f). Second, in 4-day food-deprived animals, there was a significant increase in the number of associated appetitive bites compared with the two other groups (Fig. 1g). These two changes together resulted in a significant increase in the total number of appetitive bites, as the level of food deprivation increased (Fig. 1h). Further quantitative analysis revealed that appetitive bites in 4-day food-deprived animals were organized into periods of highly regular associated bites, whereas 1-day food deprivation yielded highly irregular bite expression (Supplementary Fig. 1a,b).

We also compared the probability of a bite being generated given the biting history of the animal in the two food-deprived groups. Striking differences were seen in the plots for 1-day versus 4-day profiles (Supplementary Fig. 2a,b). In the 4-day food-deprived group, a recent bite history was associated with a high probability of short-interval future biting, consistent with a strong predictive value of recent previous biting for fast frequency feeding rhythms. By contrast, this effect was absent in 1-day food-deprived animals where bite events were essentially independent.

The behavioural results suggest that three distinct decision-making mechanisms are present in *Lymnaea*'s food-searching behaviour. Two are dependent on the animal's motivational state, one regulating the number of new appetitive bites without altering the expression of associated bites and one regulating the expression of associated bites, but not the number of new appetitive bites. In the absence of food, these two mechanisms together contribute to the graded increase in the total number of appetitive bites from fed to 4-day food-deprived state. The third mechanism is independent of the animal's motivational state and judges food presence during the appetitive bite, triggering consummatory behaviours. Next, to elucidate the neural mechanisms underlying these three distinct decision-making processes, we developed *in vitro* paradigms of these tasks.

### State-dependent food searching is expressed *in vitro*

**Figure 2:**
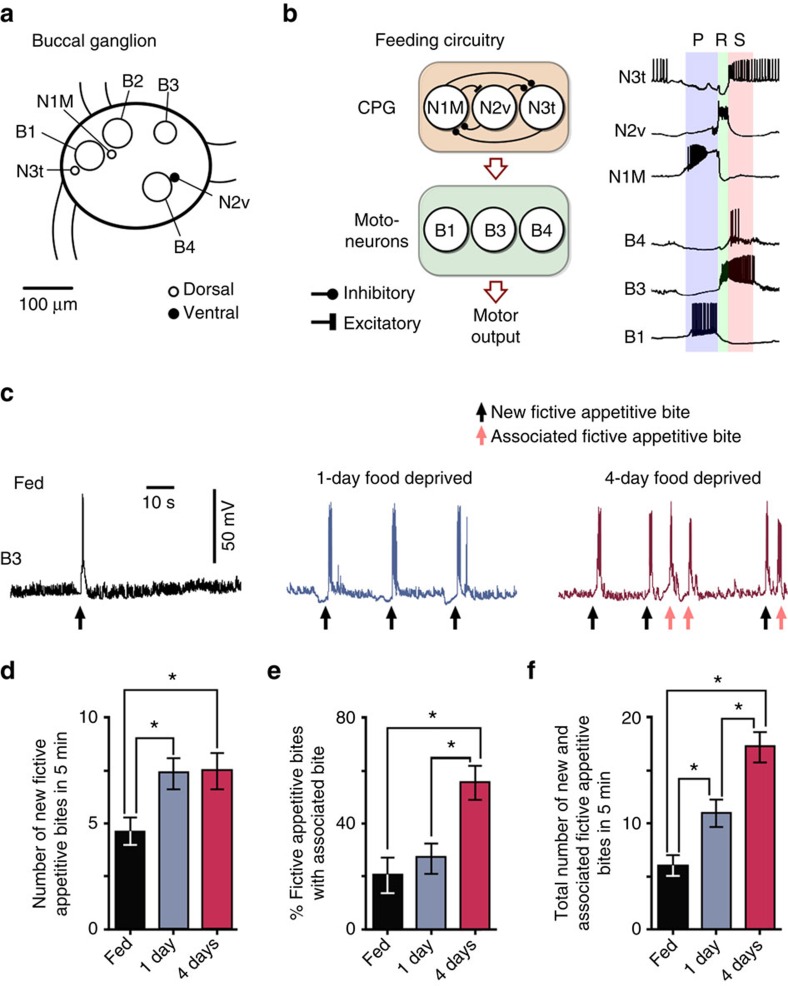
*In vitro* decision-making in *Lymnaea*. (**a**) Schematic showing one of the paired buccal ganglia in *Lymnaea*, which contains the feeding central pattern generator (CPG) interneurons (N1M, N2v and N3t) and feeding motoneurons (B1–B4). (**b**) Left: schematic diagram of the synaptic connections between CPG interneurons and their drive to the motoneurons. Right: sample recordings of the CPG interneurons and three motoneurons showing their phase of firing within a single fictive feeding motor program (P, protraction; R, rasp; S, swallow). The cycle is preceded by a period of quiescence in which N3t fires tonically. The protraction phase (Fig. 1a) starts when N1M is disinhibited from N3t. N1M activity inhibits N3t, excites N2v and drives spiking in B1. Sufficient N2v depolarization initiates the rasp phase, terminating N1M activity and initiating B3 activity. N3t recovers from inhibition from N1M and N2v, and fires a burst of spikes via post-inhibitory rebound, as does B4, initiating the swallow phase. (**c**) Representative traces of fictive appetitive bites recorded *in vitro* in isolated CNS preparations from fed (*n*=11), 1-day food-deprived (*n*=12) and 4-day (*n*=13) food-deprived animals. The B3 spiking activity is indicative of a full CPG-driven fictive appetitive bite. (**d**) Histogram showing the number of new fictive appetitive bites (mean±s.e.m.) in a 5 min observation period. One-way ANOVA, *P*<0.05. Tukey's tests, fed versus 1-day food deprived and fed versus 4-day food deprived, *P*<0.05; 1-day food deprived versus 4-day food deprived, *P*>0.05. (**e**) Histogram showing the number of fictive appetitive bites (mean±s.e.m.) that had an associated fictive bite. One-way ANOVA, *P*<0.01. Tukey's tests, 4-day food deprived versus fed and 4-day food deprived versus 1-day food deprived, *P*<0.01; fed versus 1-day food deprived, *P*>0.05. (**f**) Histogram showing the total number of fictive appetitive bites (mean±s.e.m.; new fictive appetitive and associated fictive appetitive bites). One-way ANOVA, *P*<0.0001. Tukey's tests, fed versus 1-day food deprived, *P*<0.05; fed versus 4-day food deprived, *P*<0.001; 1-day food deprived versus 4-day food deprived *P*<0.01. In panels **d**–**f**, asterisks indicate statistical significance at at least *P*<0.05.

*Lymnaea* provides an excellent model organism to examine mechanisms of decision-making at the level of individual neurons thanks to large re-identifiable cells and well-characterized circuitry^12^. The feeding network is predominantly located in the paired buccal ganglia (Fig. 2a) and feeding is controlled by a triphasic CPG, sufficient to generate a fictive form of rhythmic feeding behaviour in isolated ganglionic preparations (Fig. 2b)^3^,^13^.

First, using intracellular recording methods, we examined the expression of spontaneous fictive motor programs in isolated CNS preparations from fed, 1-day and 4-day food-deprived animals (Fig. 2c). These fictive programs are internally generated correlates of the food-searching behaviour seen *in vivo*^3^. Analogous to our behavioural observations, we found a significant increase in the number of new fictive appetitive bites in both food-deprived groups compared with the fed group (Fig. 2d). There was also a significant increase in the percentage of new cycles having associated cycles in 4-day food-deprived preparations compared with fed and 1-day food-deprived preparations (Fig. 2e), as well as a graded increase in the total number of spontaneous fictive motor programs as motivational drive increased (Fig. 2f). Thus, increased motivation serves to first augment the number of new appetitive bites and then, as food deprivation levels increase, to raise the number of associated bites.

### Changes in tonic inhibition affect food-searching decisions

**Figure 3:**
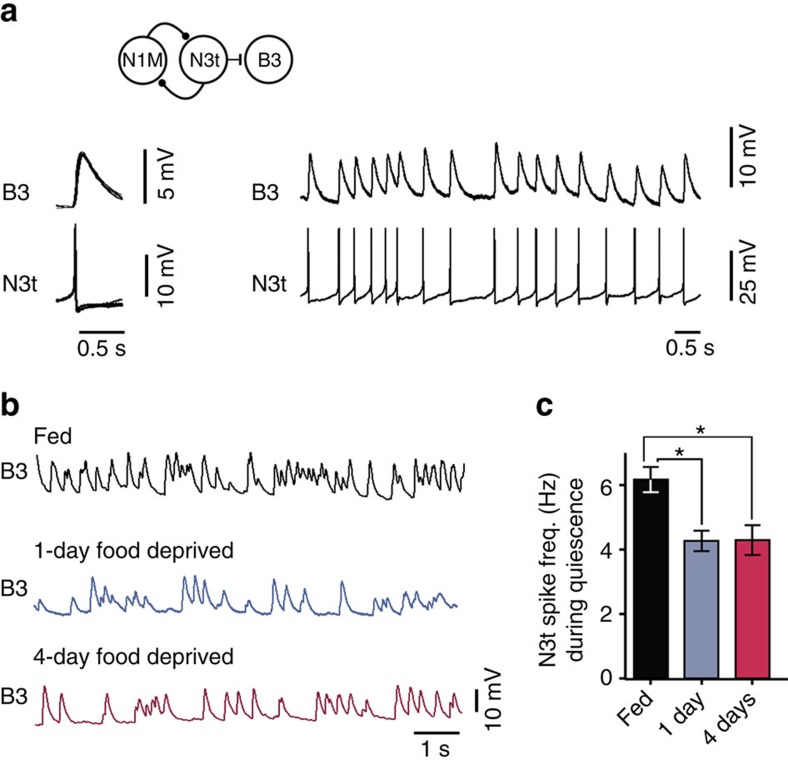
Tonic inhibitory control of feeding during quiescence. (**a**) Top: schematic of N3t's monosynaptic connections with N1M and B3. Bottom left: overlaid N3t spikes and resulting 1:1 EPSPs on B3; and bottom right: N3t tonic firing during quiescence and resulting EPSPs on B3. (**b**,**c**) Electrophysiological traces and histogram (mean±s.e.m.) of N3t-driven EPSPs on B3 in CNS preparation in fed (*n*=11), 1-day (*n*=12) and 4-day (*n*=13) food-deprived preparations. One-way ANOVA, *P*<0.01. Tukey's tests, fed versus 1 day and fed versus 4 days, *P*<0.01 (statistical significance indicated by asterisks); 1 day versus 4 days, *P*>0.05.

To identify the two different decision-making mechanisms operating in the absence of food, we set out to establish the cellular basis for the motivation-dependent effect on the number of new appetitive bites. We reasoned that one possible contributory neuron was N3t, a CPG interneuron that suppresses the feeding network through tonic inhibition during quiescence (Fig. 3a) in a motivational-state-dependent manner^3^,^14^. Therefore, we measured N3t firing rates, monitored via N3t spike-driven monosynaptic excitatory postsynaptic potentials (EPSPs) on the B3 motoneuron (Fig. 3a,b) in isolated CNS preparations made from fed, 1-day and 4-day food-deprived animals (for rationale for using B3 see Methods). We found a significant decrease in N3t firing rates between the fed preparations and both food-deprived groups (Fig. 3b,c). This suggests that the reduction in N3t firing rate in quiescence and the resulting decrease in tonic inhibition in fed versus both food-deprived groups can account for the observed increase in new fictive appetitive bites, but that a separate mechanism is responsible for the increase in associated cycles measured in the preparations from 4-day food-deprived animals.

**Figure 4:**
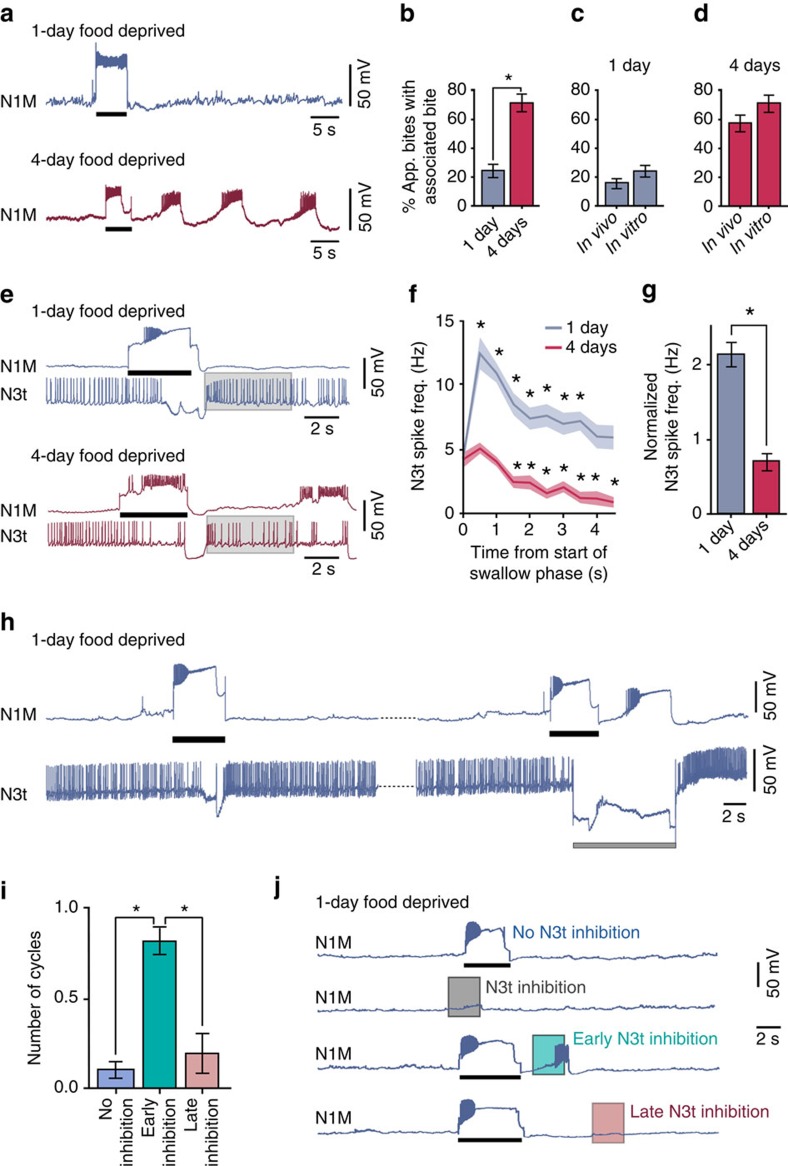
Neural mechanisms underlying motivational-state-dependent gain control of adaptive decision-making in the absence of food. (**a**) N1M activation sufficiently drives a full new fictive appetitive bite in CNS preparations in 1-day (*n*=15) and 4-day (*n*=13) food-deprived preparations. Black bars represent duration of depolarizing current injection. The current injected into N1M is needed simply to be sufficient to elicit a plateau potential, which in turn drives firing independently of the amount of injected current^47^. (**b**) Comparison of the percentage of fictive appetitive bites with an associated cycle. Unpaired *t*-test, *P*<0.0001. (**c**,**d**) Comparisons of the percentage of fictive (*in vitro*) and behavioural (*in vivo*) bites with an associated bite for 1-day and 4-day food-deprived. Unpaired *t*-test, *P*>0.05 *in vitro* versus *in viv*o, for both conditions. (**e**) Representative traces of N3t and N1M from 1-day and 4-day food-deprived preparations. Grey box shows region of N3t activity analysed. Black bars represent the duration of depolarizing current injection. (**f**) Mean spike frequency for each condition (shaded region=s.e.m.). N3t activity was significantly increased for 3.5 s compared with pre-cycle levels in 1-day food-deprived preparations. Repeated-measures ANOVA, *P*<0.0001. Dunnetts' tests, 0–0.5 to 3.0–3.5 s, *P*<0.001–0.01; 3.5–4.0 and 4.0–4.5 s, *P*>0.05 (*n*=9). Four-day food-deprived preparations showed no significant change in N3t activity for 1 s followed by a significant decrease. Repeated-measures ANOVA, *P*<0.0001. Dunnetts' tests, 0–0.5 and 0.5–1.0 s, *P*>0.05; 1.0–1.5 to 4.0–4.5 s, *P*<0.001–0.05 (*n*=7). (**g**) Comparison of N3t activity between satiety levels showing a significantly lower level in 4 days versus 1 day. Unpaired *t*-test, *P*<0.0001. (**h**) Artificial N3t hyperpolarization in 1-day food-deprived preparations sufficiently initiates an associated cycle (*n*=4). Grey bar represents the duration of hyperpolarizing current injection to N3t. (**i**) Analysis of number of cycles initiated after a fictive appetitive bite with early, late or no N3t disruption. Repeated-measures ANOVA, *P*<0.0001. Tukey's tests, no disruption versus early disruption and early versus late disruption, *P*<0.0001; no disruption versus late disruption, *P*>0.05 (*n*=9). Data are mean±s.e.m. (**j**) Representative traces of N1M during N3t disruption. Disruption initiated cycles when presented within 3.5 s after the rasp phase (third trace), but not when presented during quiescence (second trace) or outside of the 3.5 s (fourth trace). Black bars represent duration of depolarizing current injection to N1M. In **b**, **f**, **g** and **i**, asterisks indicate statistical significance.

Next, we set out to elucidate the mechanisms of the decision concerned with performing an associated bite after a new appetitive bite and how this decision is influenced by different motivational states. Here we focused on differences in the number of new appetitive bites with associated bites and their *in vitro* correlates between preparations from 1-day and 4-day food-deprived animals. We developed a fully controllable *in vitro* analogue of the spontaneous biting behaviour based on artificial activation of the CPG interneuron N1M, which is sufficient and necessary to drive fictive feeding cycles^5^. Specifically, using isolated CNS preparations (Fig. 4a), we evoked a full N1M-driven fictive feeding cycle (the *in vitro* equivalent of a new appetitive bite) and looked for the occurrence of associated cycles. Consistent with the whole-animal behaviour, the percentage of new cycles with an associated cycle was significantly lower in 1-day versus 4-day food-deprived preparations (Fig. 4b). We compared the percentage of associated cycles *in vitro* with those observed behaviourally and found no difference in either motivational state (Fig. 4c,d); as such, we refer to N1M-initiated cycles *in vitro* as fictive appetitive bites. These experiments, and those shown in Fig. 2, therefore, show that behavioural state and its effects on food-searching behaviour are preserved *in vitro* validating the use of the isolated CNS preparation for identification of the neural mechanisms of the food-searching decisions.

But, what underlies the difference in the number of associated bites under different motivational states? We reasoned that N3t activity, which is increased in the last (swallow) phase of the feeding cycle due to post-inhibitory rebound and remains increased post cycle^15^, might be a key factor. Consistent with this, we found that there was a significant increase in N3t firing rates after an N1M-triggered new fictive appetitive bite compared with pre-cycle levels in 1-day versus 4-day food-deprived preparations, where N3t firing rate actually dropped below pre-cycle levels (Fig. 4e–g). The 4-day food-deprived traces in Fig. 4e also show that similar to the experiments shown in Fig. 4a, an associated fictive appetitive bite occurs, driven by N1M. There was no statistical difference in N3t firing rates during the pre-cycle quiescence between the two levels of satiety (1-day food deprived, 4.3±0.4 Hz; 4-day food deprived, 4.2±0.6 Hz; unpaired *t*-test, *P*>0.05).

To test whether reduced levels of inhibition on the system post cycle were sufficient for the generation of associated cycles, we disrupted N3t firing in 1-day food-deprived preparations. Specifically, we injected hyperpolarizing current directly into N3t post cycle during a new fictive appetitive bite (Fig. 4h). This alone was sufficient to generate associated cycles. In a second set of experiments, we examined the relationship between the timing of N3t's inhibitory control and the effect it has on the generation of associated bites. Here we used the injection of hyperpolarizing current into a motoneuron electrotonically coupled to N3t (Supplementary Fig. 3). This allowed us to gather sufficient data without the need to co-record N1M and N3t (*n*=9) and at the same time achieve a high level of control of the timing of N3t disruption. The level of N3t disruption chosen was insufficient to initiate cycles when presented during a period of quiescence, but if disruption occurred at an early point post cycle (within ∼3.5 s), significantly more associated cycles were initiated than when no disruption was introduced or when the disruption was presented at a later time point post cycle (Fig. 4i,j). This experiment therefore identified a critical post-cycle period of ∼3.5 s during which the slowing down of N3t firing promotes the generation of associated bites.

Taken together, these results indicate that at lower motivational states, there is a post-cycle period of enhanced N3t-driven inhibition on the feeding system, which prevents the generation of associated cycles in the absence of food. However, when the motivational drive increases during increased periods of food deprivation, levels of N3t-driven inhibition are reduced and associated cycles can be generated even in the absence of food. Therefore, N3t encodes the animal's motivational state and can undergo at least two changes in firing rate, which alters the expression of fictive appetitive behaviours. In the absence of food, a decrease in N3t firing frequency during quiescence serves to increase the number of new appetitive bites (Fig. 3b,c), whereas a post cycle decrease in N3t firing frequency mediates an increase in the number of associated cycles. Thus, N3t firing rate post cycle acts as a gain control to alter the expression of associated cycles in the absence of food, allowing for a decision to be made based on internal state rather than external stimuli.

### A command-like neuron signals the presence of food

Having identified the neural mechanisms involved in the motivational-state-dependent decisions in the absence of food (the ‘motivation-encoding' circuitry), next we looked at those involved in deciding whether food was encountered during the appetitive bite, that is, a potentially independent ‘food-encoding' circuitry. To further aid this analysis, first we sought to ascertain which sensory modality was most important for the decision in intact animals.

**Figure 5:**
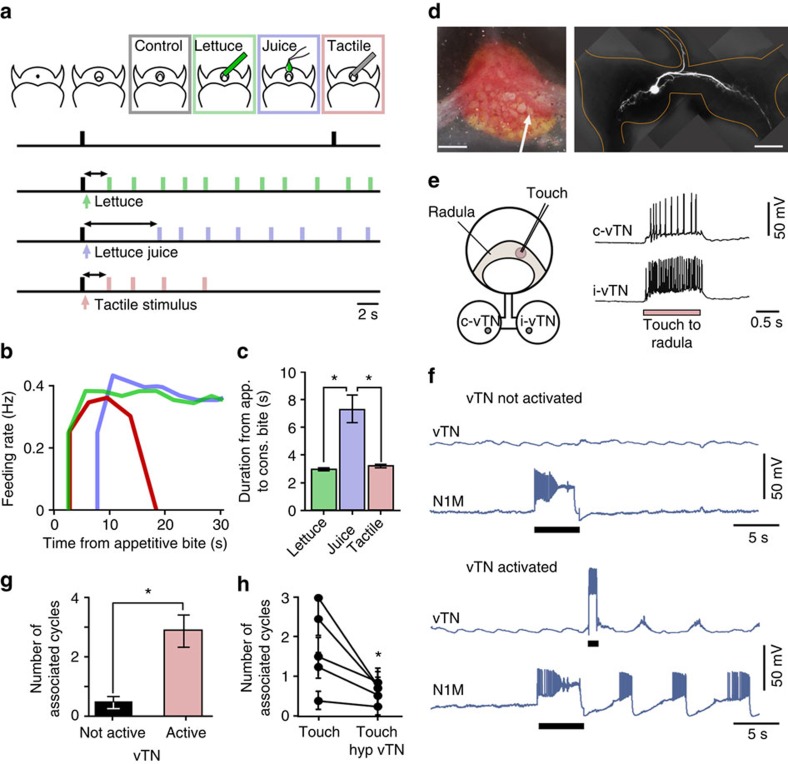
Neural mechanism of stimulus-present decision. (**a**) Sensory modality important for judgment about the presence of food during appetitive bites. Top: cartoons depict timing of application of sensory stimuli during an appetitive bite. Bottom: in the absence of food (first behavioural trace), the animal enters into quiescence. Presentation of lettuce or a tactile probe (second and fourth trace, respectively) during a bite initiates an early associated bite and further consummatory bites. Lettuce juice initiates consummatory bites with a delayed onset (third trace). (**b**) Average temporal dynamics of biting in response to stimuli presented during appetitive bite: lettuce (green), lettuce juice (blue) and tactile stimulus (red). Data in 3.5 s bins (*n*=15). (**c**) Comparison of the latency of the onset of the first associated bite. Repeated-measures ANOVA, *P*<0.0001. Tukey's tests; lettuce versus tactile stimulus, *P*>0.05; lettuce juice versus lettuce and lettuce juice versus tactile stimulus, *P*<0.001 (*n*=15). (**d**) Image of a buccal ganglion (left) indicating location of vTN (arrow). Morphology of vTN after AlexaFluor 568 dye fill (right). vTN has projections in both buccal hemiganglia and a projection into the post-buccal nerve. Scale bar, 100 μm. Morphology confirmed in *n*=11 cells. (**e**) Representative traces of the two vTN's response to tactile stimulus to the radula. vTN ipsilateral (i-) to the side of the radula touched has a larger response than the contralateral (c-) vTN. (**f**) Electrophysiological traces of N1M and vTN testing the effect of vTN activation on N1M-triggered fictive feeding cycles. In a fictive appetitive bite, vTN shows no spiking activity during a fictive appetitive bite and no associated cycles are generated (top trace). Artificial activation of vTN during the fictive appetitive bite initiates associated fictive feeding cycles (bottom trace). Black bars represent duration of depolarizing current injection. (**g**) Statistical analysis of experiments in **f**. Significantly more cycles are initiated in the presence of vTN activation than in its absence. Paired *t*-test, *P*<0.002 (*n*=13). (**h**) Analysis summarizing the effect of hyperpolarizing vTN on the number of associated fictive bites when a tactile stimulus was applied to the radula during a fictive appetitive bite. Hyperpolarizing vTN to prevent somatic spikes in response to tactile stimuli significantly reduced the number of associated cycles (Supplementary Fig. 6). Paired *t*-test, *P*<0.05 (*n*=5). All data are mean±s.e.m. In **c**, **g** and **h**, asterisks indicate statistical significance.

One-day food-deprived animals were used, since they produced more new appetitive bites than fed animals, but fewer associated bites than 4-day food-deprived animals, making the distinction between stimulus-present and stimulus-absent decisions the clearest to monitor. Animals were first tested for their food-searching behaviour in the absence of food (Fig. 5a). Only 24.0±4.3% of new appetitive bites had an associated bite in these animals, similar to those tested in the previous groups.

In a separate trial, the same animals received a piece of lettuce (solid food) during an appetitive bite (Fig. 5a). All animals produced an associated bite after the appetitive bite and the time between these (3.0±0.1 s) approached the maximum rate achievable by the CPG^5^. As such, the animal is able to make an early judgment about the presence of food during the appetitive bite and perform the appropriate action. We next tested which sensory attribute of the lettuce, either chemical or tactile, was most important for this early judgment. For this, we carried out two separate trials, one where animals received a chemical stimulus (lettuce juice) and the other involving a purely tactile stimulus (see Methods). We then measured the duration of bite onset occurring after the appetitive bite in which the stimulus was applied, and compared the results with those for solid food trials. There was a long delay between the appetitive bite and the subsequent cycle in response to the chemical stimulus alone (Fig. 5a,b; Supplementary Fig. 4), which was significantly longer than in response to the solid food (Fig. 5c). By contrast, the tactile stimulus elicited an early associated bite that closely resembled the response to lettuce (Fig. 5a–c), suggesting that the tactile properties of food provide sufficient and necessary information for the animal to make a judgment about the presence of food during the appetitive bite.

The finding that the tactile component of the food stimulus plays a decisive role during food-searching behaviour made it necessary to identify the neural mechanisms involved in the judgment about the presence of a food-signalling mechanosensory stimulus. We therefore developed a semi-intact radula CNS preparation, where the connections between the nervous system and the relevant sensory feeding structures were preserved. The objective was to identify candidate neurons that both convey tactile information from the periphery and can also initiate motor programs in a similar manner to those elicited behaviourally by the tactile stimulus.

Our search identified a novel pair of bilaterally symmetrical neurons on the ventral surface of the buccal ganglia (ventral trigger neuron, vTN, Fig. 5d) with extensive projections in both the ipsi- and contralateral ganglia (Fig. 5d) and the ability to excite N1M to trigger a full fictive feeding cycle (Supplementary Fig. 5). vTNs responded to tactile stimulation of the radula with a burst of spikes (Fig. 5e), with larger responses in the neuron ipsilateral to the region of the radula being stimulated.

Next, we tested whether vTN had a role in the decision about the presence of a mechanosensory stimulus during an appetitive bite by artificially activating it in a fictive appetitive bite in isolated CNS preparations from 1-day food-deprived animals. We observed that an N1M-driven new fictive appetitive bite did not result in somatic spiking activity in vTN (Fig. 5f) and, as demonstrated previously, there were no associated feeding cycles. However, in the same preparation, brief artificial activation of a single vTN after a fictive appetitive bite was sufficient to initiate a significant number of fictive bite programs (Fig. 5f,g). A comparison between the numbers of associated cycles *in vitro* in vTN-activated trials versus tactile stimulus trials *in vivo* revealed that there was no significant difference (vTN-activated trials, 2.8±0.6 (*n=*13); tactile stimulus *in vivo*, 3.9±0.4 (*n=*15); unpaired *t*-test, *P*>0.05). Taken together, these findings implicate vTN as a key neuron in the decision-making pathway for establishing the presence of food.

To test the necessity of vTN directly, next we carried out experiments in radula CNS preparations, where artificial activation of vTN was replaced by its behavioural analogue—tactile stimulation of the radula—during a fictive appetitive bite (‘stimulus-present' trial, see Supplementary Methods). We quantified the number of associated bites under two conditions, one with vTN at resting potential and the second where vTN was hyperpolarized to prevent somatic spikes during the tactile stimulation. In the latter case, there was a significant reduction in the number of cycles triggered by the tactile stimulus (Fig. 5h; Supplementary Fig. 6) compared with the former. This finding strongly implicates vTN as a key neuron in the pathway that reports the presence of food during an appetitive bite and the triggering of associated bites. The lack of vTN activity during N1M-initiated cycles also provides a possible mechanism by which the stimulus-absent decision is made, based simply on the lack of activity in the decision neuron providing no drive to the system in the absence of sensory information during the appetitive bite.

Taken together, vTN activity therefore provides a simple mechanism by which the animal judges the presence or absence of a key food-signalling sensory stimulus and responds appropriately. On the basis of the above results, we will refer to cycles in which vTN was activated by current injection as fictive ‘stimulus-present' trials, and those in which vTN was not activated as fictive ‘stimulus-absent' trials.

### Motoneurons fire in energy-saving mode during food searching

**Figure 6:**
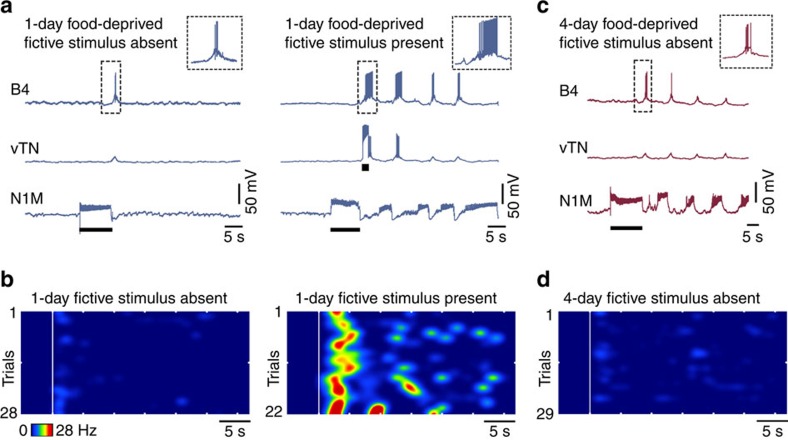
Neural correlates of energy saving during food searching and consummatory feeding. (**a**) Electrophysiological traces of neurons B4, vTN and N1M in an experiment testing the impact of artificial activation of vTN (fictive stimulus present) on N1M-triggered fictive feeding. The two (left and right) sets of three co-recorded traces show examples with a fictive stimulus-absent (left) and a fictive stimulus-present (right) trial from a preparation from a 1-day food-deprived animal (*n*=9). Boxed areas show an expanded trace of B4 activity. B4 firing frequency in fictive stimulus-absent trials (1.7±0.5 Hz) was significantly lower versus both the first (9.7±1.1 Hz) and associated cycles (6.7±1.8 Hz) of the fictive stimulus-present trials. Repeated-measures ANOVA, *P*<0.001; Tukey's tests, first fictive stimulus-present cycle versus first fictive stimulus-absent cycle, *P*<0.001; first fictive stimulus-present associated cycle versus first fictive stimulus-absent cycle, *P*<0.05; the first and associated cycles of the stimulus-present trials were not significantly different (*P*>0.05). Black bars represent the duration of depolarizing current injection. (**b**) Smoothed heat plots showing B4 firing frequency in multiple trials for fictive stimulus absent (left) and fictive stimulus present (right) in 1-day food-deprived animals (*n*=9 independent preparations). Colour coding represents frequency. Each trial is aligned to the onset of the first N2 phase indicated by a white vertical line. (**c**) B4, vTN and N1M activity recorded during a fictive stimulus-absent trial in a preparation from a 4-day food-deprived animal (*n*=11). Boxed area shows an expanded trace of B4 activity. B4 firing frequency in the first fictive appetitive cycles (1.2±0.4 Hz) was not significantly different from that measured in the fictive stimulus-absent trials in 1-day food deprived animals (*n*=9). Unpaired *t*-test *P*>0.05. Black bars represent duration of depolarizing current injection into N1M. (**d**) Smoothed heat plots showing B4 firing frequency in multiple fictive stimulus-absent trials in 4-day food-deprived animals (*n*=11 independent preparations).

*Lymnaea* relies on the generation of energetically costly feeding cycles for both its food-searching and consummatory behaviours. As such, we hypothesized that reduced motor pattern expression in the former versus the latter might provide a form of energy saving until the presence of food is established. To test this idea, we used the isolated CNS preparation to compare motoneuronal firing activity in the phases following the onset of fictive feeding cycles corresponding to appetitive and consummatory bites, respectively. Activity of B10, a major rasp-phase motoneuron of the feeding system^9^, was very comparable in fictive stimulus-absent versus fictive stimulus-present trials (fictive stimulus absent, 10.7±1.2 Hz; fictive stimulus present, 11.3±1 Hz; paired *t*-test, *P*>0.05 (*n=*5); Supplementary Fig. 7). By contrast, activity in B4, a key swallow-phase motoneuron^9^, was strikingly different between the two fictive behaviours. In the first cycle of the fictive stimulus-absent trials, B4 firing frequency was significantly lower versus both the first and associated cycles of the fictive stimulus-present trials (Fig. 6a,b), whereas there was no significant difference between the first and the associated cycles of the stimulus-present trials. Moreover, the lower rate of B4 firing in the absence of fictive tactile stimulus persisted in 4-day food-deprived animals (Fig. 6c,d), even though the feeding CPG generated more cycles of activity after the initial activation of N1M had triggered the first cycle. Our findings are consistent with the idea that rasp-phase expression is critically invariable between appetitive and consummatory modes, but swallow-phase activity is attenuated in food searching and significantly upregulated during consummatory biting. We suggest that limiting engagement of the whole swallow-phase network during food-searching behaviour—corresponding to the repeated, coordinated activation of tens of motoneurons and muscles—would be significant with regard to energy savings.

Notably, vTN showed no activity in the fictive stimulus-absent trials in isolated CNS preparations from 1- or 4-day food-deprived animals (Fig. 6a,c), confirming that vTN is only involved in the activation of the feeding CPG in the stimulus-present decision (Fig. 6a, right panels) when full B4 bursts are generated. When vTN is activated during the first cycle, B4 fires in a manner representing fictive consummatory bites, while in the absence of vTN activity, B4 firing represents fictive appetitive bites. Thus, fictive stimulus-present and stimulus-absent judgments can be distinguished *in vitro* based on the output of specific motoneuron types.

### Parallel pathways encode motivation and food presence

**Figure 7:**
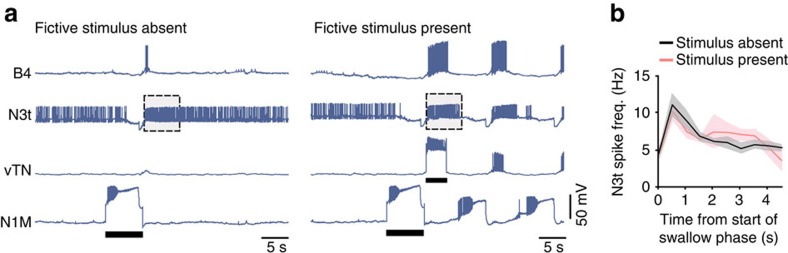
Parallel-independent pathways encode motivation and food presence in decision-making. (**a**) Representative sets of co-recorded traces of B4, N3t, vTN and N1M in a fictive stimulus-absent (left) and a fictive stimulus-present trial (right). N3t activity was analysed post cycle in the two conditions. The grey box represents the time window of N3t activity post cycle analysed and compared with pre-cycle levels in **b**. Black bars represent the duration of depolarizing current injection. (**b**) Line plots of average N3t spike frequency in fictive stimulus-absent and fictive stimulus-present trials with shaded region showing s.e.m. N3t firing rate was measured for 5 s pre-cycle and binned into 0.5 s bins. N3t firing rates for 4.5 s post cycle were binned into 0.5-s bins. In both **a** and **b**, note the lack of direct synaptic connections between N3t and vTN.

The identification of the two key neuron types, vTN and N3t, involved in the decision-making processes provided the opportunity to test the interactions of decision neurons at the point that a decision is made to generate associated cycles. N3t activity was analysed in 1-day food-deprived isolated CNS preparations during fictive stimulus-absent and fictive stimulus-present trials to determine how it was affected by the stimulus-present decision (Fig. 7a,b). We compared N3t activity between the two trials and found no significant difference (fictive stimulus absent, 1.9±0.2; fictive stimulus present, 1.9±0.2; paired *t*-test, *P*>0.05, *n=*5). Therefore, in the fictive stimulus-present decision, N3t activity increases as in the fictive stimulus-absent trial, yet vTN provides a sufficient amount of excitation to the system to overcome the high levels of inhibition from N3t. This suggests that the two decision processes work independently of each other, allowing the system to encode information about motivational state and, in parallel, the presence or absence of food-signalling sensory stimuli during the goal-directed behaviour.

## Discussion

In this study, we describe a neural system—the most parsimonious that we are aware of—for interlinked perceptual and state-dependent decision-making in a goal-directed behaviour. The core decision-making circuit consists of just two identified neuron types with N3t acting as a gain controller encoding the animal's motivational state (Figs 1, 2, 3, 4) and vTN encoding the presence or absence of relevant sensory inputs (Fig. 5). The current study builds on previous knowledge on the feeding circuitry in *Lymnaea*, but takes it significantly further by analysing its functioning in the context of goal-directed decision-making that has been observed in both simple and higher animals, but so far not understood in sufficient cellular detail in any system.

**Figure 8:**
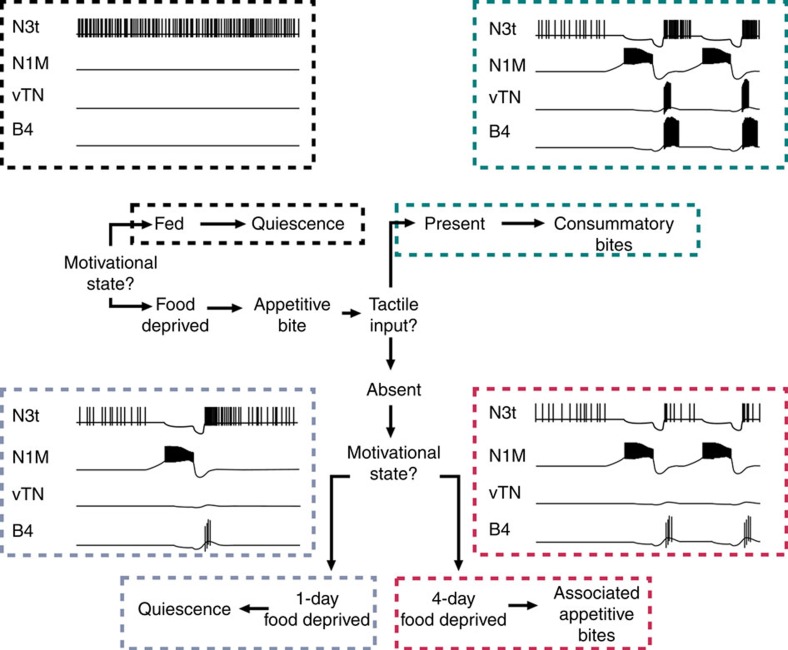
Schematic of decision-making during food searching in *Lymnaea*. On the basis of their feeding-related internal state (fed or food-deprived), *Lymnaea* decides whether to perform an appetitive bite. Levels of tonic inhibition on the system from the multifunctional ‘motivation-encoding' N3t interneuron determine whether a bite is initiated or not (decision mechanism 1). At low motivational levels (fed animals), tonic N3t firing rates are high (black box), preventing the expression of a new appetitive bite (‘Quiescence'). At higher motivational levels (1-day or 4-day food-deprived animals), N3t firing during quiescence decreases and a new appetitive bite is performed, driven by the activation of the CPG neuron N1M. On performing the food-searching behaviour, *Lymnaea* judges the presence or absence of a potential food during the appetitive bite and performs an appropriate response based on both external and internal cues. Spike activity of the ‘food-sensing' vTN signals the presence of solid food (decision mechanism 2) and triggers a bout of consummatory bites, characterized by strong motoneuronal bursting activity (B4) in the swallow phase (teal box, ‘Consummatory bites'). In the absence of potential food during the appetitive bite, vTN remains silent and a further decision is made based on the animal's motivational state (decision mechanism 3). At lower motivational levels (1-day food deprivation), *Lymnaea* will typically enter back into a period of quiescence (blue box, ‘New appetitive bite with no associated bite'). This is due to higher levels of in-cycle inhibition on the system from N3t. At higher motivational states (4-day food deprivation), associated appetitive bites are performed due to lower levels of in-cycle inhibition from N3t (red box, ‘New appetitive bite with associated bite'). In the absence of tactile input, both new and associated appetitive bites are characterized by low levels of B4 motoneuronal activity in the swallow phase, incorporating an energy-saving mechanism in the absence of food.

**Figure 9:**
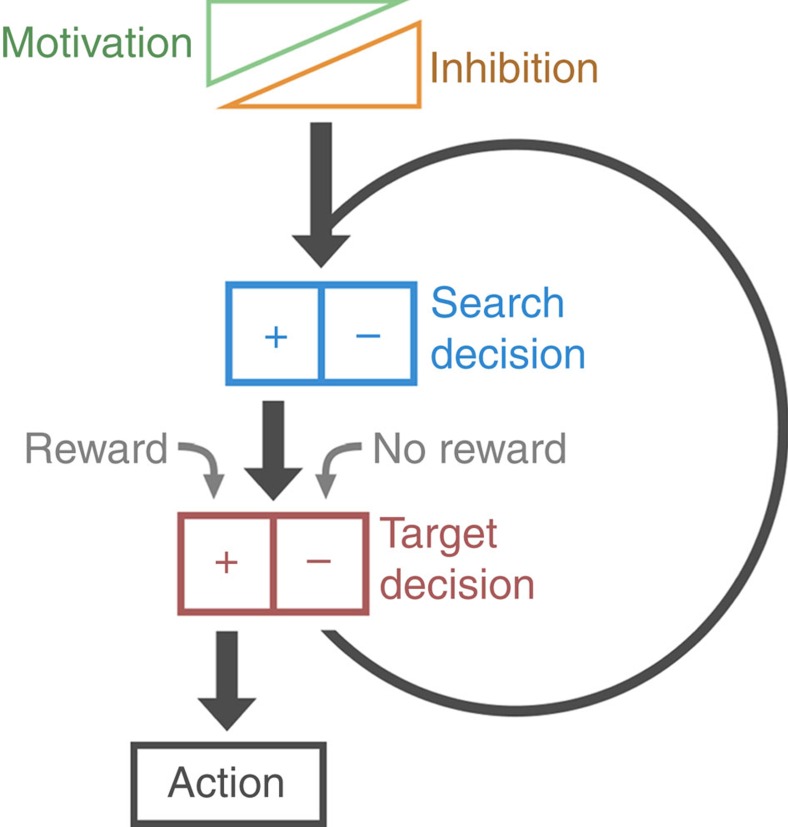
Generalized scheme for goal-directed decision-making. A highly simplified cartoon schematic illustrating key principles of the decision-making pathway characterized here. The decision to search is regulated by tonic inhibition, the magnitude of which is inversely correlated with motivational drive. Search has two outcomes depending on target decision: activation of the full behavioural sequence if reward is present or a return to the search decision step if unrewarded. In this way, a motivated animal sustains a high search intensity, maximizing the chance of reward.

The mechanisms of goal-directed decision-making occurring during food-searching behaviour in *Lymnaea*, and the role of the two types of decision neurons, vTN and N3t, are summarized in Fig. 8. Due to a high level of tonic firing in N3t, satiated (fed) *Lymnaea* show very few, if any, appetitive bites (‘quiescence'). Food-deprived animals also undergo periods of feeding quiescence, but during these periods the tonic firing rate of N3t is lower compared with fed animals, increasing the expression of new appetitive bites. When *Lymnaea* encounters food during an appetitive bite (‘stimulus present'), consummatory bites are triggered, which are characterized by high-frequency burst firing of the swallow-phase motoneuron B4. Activation of a single command-type neuron, vTN, is both sufficient and necessary for this simple perceptual decision. In the absence of food, vTN remains silent and a state-dependent decision is made, again encoded by the firing frequency of N3t (ref. 3). In 1-day food-deprived animals, where N3t's post-cycle inhibitory influence on the network is stronger, an appetitive bite is seldom followed by an associated bite and the feeding system re-enters into quiescence. By contrast, in 4-day food-deprived animals, post-cycle N3t activity is downregulated, providing a state-dependent mechanism for increasing associated appetitive bites even in the absence of sensory information. Together, the two N3t-dependent mechanisms result in a greater increase in the total number of appetitive bites, and thus the likelihood of locating a food source, as a function of the level of food deprivation. Potentially general mechanisms of goal-directed decision-making based on the state-dependent inhibition and reward-induced activation of the same behaviour-generating network are shown in Fig. 9.

In most previous studies that used invertebrate models to examine neuronal mechanisms underlying types of state-dependent decision-making, the basis of the decision is a selection of one type of behaviour over another^16^,^17^,^18^,^19^,^20^,^21^,^22^. Our study, however, demonstrates a decision to perform a behaviour versus not to perform it, depending on both the presence or absence of relevant sensory input and the motivational state of the animal. The presence of food always triggered consummatory bites in *Lymnaea*, regardless of the level of satiety. We established that the key sensory modality involved in deciding food presence was mechanosensation and the decision neuron was vTN. The motivational state encoded by N3t only becomes important in the absence of food when food-deprived animals performed more appetitive bites compared with well-fed ones, providing a cellular mechanism for active decision-making in the absence of sensory stimuli.

A number of studies have considered how an animal's perception of a stimulus alters, based on motivational and contextual states, and the neural mechanisms involved in such decisions^16^,^23^,^24^,^25^,^26^. Notably, in the stimulus-present decision made by *Lymnaea*, the animal's motivational state does not alter the decision to perform consummatory behaviours, presumably because food is presented during an appetitive bite performed with the goal of locating food, so the animal is ‘primed' to respond to a food-signalling stimulus. However, we identified that decision-making in the absence of food was dependent on the animal's motivational state.

There are interesting parallels between operational features of this simple network and cellular mechanisms of feeding-related decision-making in mammals. For example, food deprivation-sensitive neurons have been identified in the hypothalamus of the mouse^27^. Artificial upregulation of activity in a subset of these neurons not only elicits voracious eating in fed animals^28^ but also increases the animal's motivational drive to obtain food^27^,^29^, suggesting that, similar to N3t in *Lymnaea*, these neurons not only play a role in consummatory behaviours but also in setting motivational-state and subsequent appetitive behaviours. Furthermore, and again analogous to N3t, these neurons appear to exert their control on food-related decision-making via inhibitory synaptic connections^28^. Feeding behaviour in *Drosophila* has also been shown to be under the influence of tonic levels of inhibition^25^, suggesting that this may be a common underlying mechanism involved in feeding-related decision-making. Finally, vTN's role in transforming sensory inputs into an appropriate motor output bears similarities to other ‘go'-type neurons found in both invertebrates and vertebrates^30^,^31^,^32^.

Unlike previous cellular studies on decision-making, we show that a remarkable feature of the mechanisms identified here is the incorporation of energy-saving strategies that limit both unnecessary action potential generation and associated behavioural expression (Fig. 6). Specifically, we see a sixfold reduction in the firing frequency of a swallow-phase motoneuron in appetitive versus consummatory bites. The significance of this is that the swallow phase engages high-frequency burst activity in >20 coupled neurons recruiting muscle groups associated with movement of the buccal mass and oesophageal structures^12^ and as such, with profound implications for energy expenditure. In the blowfly, downregulation of activity in neurons involved in non-essential behaviours has been reported to occur in a motivational-state-dependent manner^33^. In *Lymnaea*, there is a sound biological rationale for energy saving during swallowing; this is the final phase of the feeding cycle and so its full expression is only necessary once food has been located. In other words, the system opportunistically adapts its output by attenuating activity in a phase that is not essential for the full expression of the goal-oriented behaviour. Consistent with this idea, we note that the first two phases of the feeding cycle, protraction and rasp, which require full activation to maximally protrude the radula structure and thus detect the presence of food, retain the same expression properties regardless of the nature of the bite. Thus, this appears to be an efficient energy-saving strategy limiting behavioural expression where possible, but not at the expense of the most effective read-out of the presence of food.

## Methods

### Animal maintenance

Animals were kept in groups in large holding tanks containing Cu^2+^-free water at 20 °C on a 12:12-h light–dark regime. The animals were fed lettuce three times a week and a vegetable-based fish food (Tetra-Phyll; TETRA Werke, Melle, Germany) twice a week. Animals were transferred to smaller holding tanks before experimenting. Animals were either fed *ad libitum* or were food deprived for either 1 or 4 days before either behavioural or electrophysiological experiments.

### Behaviour

*Lymnaea*'s behaviour was observed by placing them in a custom-built behavioural chamber filled with Cu^2+^-free water. The chamber held the animal on the surface of water allowing for the application of sensory stimuli to the mouth of the snail while being able to fully observe movements of the feeding structures. *Lymnaea* often search for food/feed while moving across the surface of water; therefore, this design allowed for the observation of their food-searching and feeding behaviour in a behaviourally relevant manner. All behavioural experiments were videoed and analysed using ImageJ and Spike2 software.

All animals were left to acclimatize for 10 min prior to testing. In the first set of behavioural experiments animals of different levels of satiety (fed, 1-day and 4-day food deprived) were observed for 30 min, and their appetitive-biting behaviour was recorded in the absence of sensory stimuli. During all behavioural experiments, bites that occurred within 6 s of the previous bite were considered associated with the previous bite. The rationale for this was that *Lymnaea* can perform a bite every 2–5 s in the presence of a sensory stimulus^34^,^35^,^36^,^37^. Thus, a 6 s value was chosen in this study to ensure that no associated bites were missed. Any bite that occurred outside the 6 s window after an appetitive bite was classified as a new appetitive bite. At the end of the 30 min trial, all animals were presented with a piece of lettuce (2 mm by 1 mm) during the first appetitive bite performed. Bites in the presence of lettuce (or any presented sensory stimuli) were classified as consummatory bites. The number of bites within 30 s of the appetitive bite were counted and compared between satiety levels.

To examine the sensory modality important for the judgment of the presence of food during the appetitive bite, 15 animals were tested under five conditions. Animals were separated into five groups of three and starved for 1 day before testing. Animals were left to acclimatize in the behavioural chamber for 10 min before testing. Each trial lasted up to 50 min. In the first condition, their appetitive-biting behaviour was observed in the absence of sensory stimuli. In the second condition, animals received two pieces of solid lettuce during appetitive bites, the first piece was presented during the first appetitive bite after the acclimatization period and the second during an appetitive bite at least 20 min after the first presentation of lettuce. In the third condition, ∼10 μl of a homogenate of lettuce, referred to as lettuce juice, was applied to the animal's mouth during an appetitive bite. In the fourth condition, a tactile stimulus was placed in the animal's mouth as in the solid lettuce trial. The tactile probe consisted of a 1-ml syringe whose tip had been heated and pulled into a fine point. In the fifth trial, lettuce juice was applied to the animal's mouth as in the third trial, but in the absence of an appetitive bite.

After each trial, the animal was placed in a fresh container of Cu^2+^-free water with lettuce leaves in it. Animals were allowed to feed *ad libitum* for a day after each trial. They were then food deprived for a single day before the next trial. To ensure that the order of the trials did not affect the results, each group of animals performed a different trial on each test day.

### Preparations and electrophysiological methods

*In vitro* experiments were carried out using a previously described isolated CNS preparation^38^ and a novel semi-intact radula CNS preparation (Fig. 4e). In the first type of preparation, the buccal ganglia were twisted to allow the simultaneous recording from vTN on the ventral side of one hemi-ganglion and N3t, N1M and motoneurons on the dorsal side of the other hemi-ganglion. The second type of preparation was developed as part of this study because the behavioural experiments demonstrated that the sensory modality important for the early judgment about the presence of food during an appetitive bite was its tactile properties. In the behavioural experiments, the toothed radula is the structure that comes into contact with the tactile stimulus, as it is retracted back into the mouth. In other molluscs, neurons located in the buccal ganglia have been identified, which respond to tactile stimulation of the radula^39^. To identify similar neuron types in *Lymnaea*, we systematically tested neuronal responses to radula tactile input using the radula CNS preparation. Briefly, this preparation consisted of the entire CNS connected to the buccal mass via the post-buccal nerve. An incision was made in the buccal mass from the oesophageal opening to the dorsal mandible and pinned so as to expose the odontophore and the toothed radula.

Preparations were perfused with normal saline containing 50 mM NaCl, 1.6 mM KCl, 2 mM MgCl_2_, 3.5 mM CaCl_2_ and 20 mM HEPES buffer in water. Intracellular recordings were made using sharp electrodes (10–40 MΩ) filled with 4 M potassium acetate. NL 102 (Digitimer Ltd) and Axoclamp 2B (Axon Instrument, Molecular Device) amplifiers were used, and data were acquired using a micro 1401 Mk II interface and analysed using Spike2 software (Cambridge Electronic Design, Cambridge, UK). To determine the response of neurons to tactile stimulation of the radula, a fire-polished pipette was used as a probe. The probe was mounted on a micromanipulator allowing for tactile stimulation of specific regions of the radula.

### Neurons recorded in the experiments and their identification

Buccal ganglia motoneurons B3, B4 and B10 were recorded to monitor CPG-driven fictive feeding. Briefly, B10 is a rasp-phase motoneuron, B3 is a rasp/swallow-phase motoneuron, and B4 and B8 are swallow-phase motoneurons. In addition, B3 was recorded to monitor N3t firing activity. N3t is the only source of discrete short-latency EPSPs that can be recorded in B3 during feeding quiescence^15^, and the use of B3 to monitor N3t firing without the need to impale and hold this small and fragile interneuron for extended periods is well documented^3^,^14^,^40^. Moreover, B3 is a glandular motoneuron^41^ and unlike most of the motoneurons innervating buccal musculature it does not have reciprocal electrical connections with any of the CPG neurons^9^; thus, during bursting it cannot influence their activity. Motoneuron B8 was recorded and hyperpolarized to stop N3t firing via its electrotonic synaptic connection. These motoneurons can be identified by their size, location and synaptic inputs during fictive feeding cycles^9^,^12^,^38^.

The CPG interneurons N1M and N3t, which have reciprocal inhibitory connections, also were recorded and their firing activity manipulated through current injection. N1M has an endogenous plateau potential^42^ and artificial activation of a single N1M is sufficient to elicit a full fictive feeding cycle. N3t fires tonically and can be identified via its monosynaptic excitatory connection with B3 (ref. 15).

vTN is a newly identified neuron located on the ventral surface of the buccal ganglia close to the buccal commissure. It can be identified by its white colour and location with respect to the buccal commissure. Tactile stimulation of the lateral edges of the radula and the distal region of the odontophore (the structure on which the radula is positioned) in a radula CNS preparation elicits somatic spikes in vTN. In experiments with semi-intact radula CNS preparations, the cerebral–vental neurons CV1 and CV3 were stimulated and recorded, respectively, to test the importance of vTN for the stimulus-present decision. These neurons were identified based on their known firing patterns and effects on the feeding CPG^43^,^44^.

### Iontophoretic dye filling of neurons

Neurons were filled with a fluorescent dye allowing the morphology of the cell to be determined. Microelectrodes were filled with either 5(6)-carboxyfluorescein or AlexaFluor 568 (Molecular Probes). Cells were filled iontophoretically using a pulse generator that applied regular interval negative square current pulses into the neuron for >30 min. The preparation was then left overnight at 4 °C. Images of the neurons were taken using a digital camera (Andor Ixon EMCCD) mounted on a Leica stereomicroscope, using 470-nm excitation (CoolLED) and 520/10 emission or using a laser scanning confocal microscope (Olympus BX51WI, FV300) with 488-nm Argon or 543-nm HeNe laser for excitation and 520/10 nm or 600LP nm for emission.

### *In vitro* paradigm and classification of associated cycles

Fictive feeding programs were recorded from a variety of motoneurons and interneurons. Fictive cycles were recorded as spontaneously generated cycles or elicited via N1M activation. Spontaneous fictive appetitive bites were recorded over a 5 min period. N1M-triggered fictive feeding cycles were generated by artificially activating a single N1M by injecting depolarizing current sufficient to initiate a plateau potential in the neuron^42^. Current injection was maintained until the onset of the N2 phase, which in turn terminates the N1M plateau potential and can also be seen by the appropriate synaptic inputs to co-recorded motoneurons^38^. As fictive feeding programs are slower *in vitro* than those seen *in vivo*^45^, a separate criterion was set to classify associated fictive feeding cycles. N1M-driven cycles are ∼9 s long^46^; we therefore classified a cycle as associated if it occurred within 9 s of the onset of the N2 phase of the previous cycle.

N3t activity recorded from B3 was analysed in the first 10 s of quiescence, 2 min after impaling a B3. N3t activity in fictive appetitive bites was analysed by recording N3t firing rates for 5 s before initiation of a fictive appetitive bite and binning the data into 0.5 s bins. N3t firing rates for 4.5 s post cycle were binned into 0.5 s bins. Comparisons between N3t firing rates in 1-day and 4-day food-deprived and stimulus-present and stimulus-absent trials were compared by normalizing N3t firing rates for 3.5 s post cycle with 5 s pre-cycle and comparing between groups. The 3.5 s time window was chosen since this was the duration of increased N3t firing rates in one-day food-deprived preparations.

N3t disruption experiments were performed by injecting hyperpolarizing current directly into N3t or into the B8 motoneuron, which is electrically coupled with N3t (Supplementary Fig. 3). In the latter case, a level of B8-induced disruption was identified which, when presented during a period of quiescence, was insufficient to elicit fictive feeding cycles. The average duration of the stimulus was 2.1±0.1 s and current injection was −6.3±1 nA. The stimulus was repeated three times during quiescence in each experiment. The same hyperpolarizing current stimulus was applied to B8 in a fictive appetitive bite within 3.5 s post cycle or after 3.5 s post cycle.

Fictive stimulus-present trials were those in which vTN was artificially activated to fire somatic spikes. In these trials, vTN was artificially depolarized to fire at a rate of 20–30 Hz. This was used because it corresponded to the rate at which vTN fired in response to tactile stimulation of the radula in the radula CNS preparation. vTN was stimulated to fire in the late rasp/early swallow phase (late N2/early N3), as behavioural observations showed that the radula came into contact with the tactile probe during the retraction of the radula into the mouth. Fictive stimulus-absent trials were those in which vTN was not activated by current injection after a new fictive appetitive bite. ‘Stimulus-present' trials were those in which a tactile stimulus was applied to the radula after a new fictive appetitive bite in a radula CNS preparation, whereas in a ‘stimulus-absent' trial, the radula was not stimulated following a fictive bite.

B4 spike frequency was recorded over a set period of 2 s from the end of the N2 phase during the first initiated fictive appetitive bite and compared between fictive stimulus-absent and stimulus-present trials in 1-day food-deprived and 4-day food-deprived preparations. Heat plots of multi-trial B4 spike activity occurring over 30 s starting from the N2 phase of the first initiated fictive appetitive bite were generated in Matlab (MathWorks) using the ‘surf' function. Spike frequency was encoded by a false colour look-up table and smoothed with a Gaussian filter.

### Statistical analysis of data

Data were analysed using GraphPad Prism 5 (GraphPad Software) and expressed as mean±s.e.m. Each ‘*n*' represents an individual preparation. Normality was tested using the D'Agostino and Pearson omnibus normality test. Two-group statistical comparisons were performed using two-tailed *t*-test statistics (either paired or unpaired as stated in the text). Data with more than two groups were first analysed using either a one-way ANOVA or one-way repeated-measures ANOVA. Subsequent comparisons were performed using Dunnett's or Tukey's multiple comparison tests. The significance level was set at *P*<0.05.

### Data availability

The behavioural and electrophysiological data that support the findings of this study are available in ‘figshare' with the identifier https://dx.doi.org/10.6084/m9.figshare.3249310.

## Additional information

**How to cite this article:** Crossley, M. *et al*. A two-neuron system for adaptive goal-directed decision-making in *Lymnaea*. *Nat. Commun.* 7:11793 doi: 10.1038/ncomms11793 (2016).

## Supplementary Material

Supplementary Information Supplementary Figures 1-7, Supplementary Methods and Supplementary References

## References

[b1] Gold J. I. & Shadlen M. N. The neural basis of decision making. Annu. Rev. Neurosci. 30, 535–574 (2007). 17600525 10.1146/annurev.neuro.29.051605.113038

[b2] Rangel A. & Hare T. Neural computations associated with goal-directed choice. Curr. Opin. Neurobiol. 20, 262–270 (2010). 20338744 10.1016/j.conb.2010.03.001

[b3] Staras K., Kemenes I., Benjamin P. R. & Kemenes G. Loss of self-inhibition is a cellular mechanism for episodic rhythmic behavior. Curr. Biol. 13, 116–124 (2003). 12546784 10.1016/s0960-9822(02)01435-5

[b4] Pirger Z. . Interneuronal mechanism for Tinbergen's hierarchical model of behavioral choice. Curr. Biol. 24, 2018–2024 (2014). 25155505 10.1016/j.cub.2014.07.044 PMC4159561

[b5] Kemenes G., Staras K. & Benjamin P. R. Multiple types of control by identified interneurons in a sensory-activated rhythmic motor pattern. J. Neurosci. 21, 2903–2911 (2001). 11306642 10.1523/JNEUROSCI.21-08-02903.2001 PMC6762524

[b6] Straub V. A. & Benjamin P. R. Extrinsic modulation and motor pattern generation in a feeding network: a cellular study. J. Neurosci. 21, 1767–1778 (2001). 11222666 10.1523/JNEUROSCI.21-05-01767.2001 PMC6762967

[b7] Brierley M. J., Yeoman M. S. & Benjamin P. R. Glutamatergic N2v cells are central pattern generator interneurons of the lymnaea feeding system: new model for rhythm generation. J. Neurophysiol. 78, 3396–3407 (1997). 9405553 10.1152/jn.1997.78.6.3396

[b8] Kemenes G. & Elliott C. J. Analysis of the feeding motor pattern in the pond snail, *Lymnaea stagnalis*: photoinactivation of axonally stained pattern-generating interneurons. J. Neurosci. 14, 153–166 (1994). 8283231 10.1523/JNEUROSCI.14-01-00153.1994 PMC6576837

[b9] Staras K., Kemenes G. & Benjamin P. R. Pattern-generating role for motoneurons in a rhythmically active neuronal network. J. Neurosci. 18, 3669–3688 (1998). 9570798 10.1523/JNEUROSCI.18-10-03669.1998 PMC6793163

[b10] Yeoman M. S., Brierley M. J. & Benjamin P. R. Central pattern generator interneurons are targets for the modulatory serotonergic cerebral giant cells in the feeding system of Lymnaea. J. Neurophysiol. 75, 11–25 (1996). 8822538 10.1152/jn.1996.75.1.11

[b11] Rose R. M. & Benjamin P. R. The relationship of the central motor pattern to the feeding cycle of *Lymnaea stagnalis*. J. Exp. Biol. 80, 137–163 (1979). 501275 10.1242/jeb.80.1.137

[b12] Benjamin P. R. Distributed network organization underlying feeding behavior in the mollusk Lymnaea. Neural. Syst. Circuits 2, 4 (2012). 22510302 10.1186/2042-1001-2-4 PMC3350398

[b13] Marra V., O'Shea M., Benjamin P. R. & Kemenes I. Susceptibility of memory consolidation during lapses in recall. Nat. Commun. 4, 1578 (2013). 23481386 10.1038/ncomms2591 PMC3615469

[b14] Marra V. . Role of tonic inhibition in associative reward conditioning in *Lymnaea*. Front. Behav. Neurosci. 4, 161 (2010). 20877424 10.3389/fnbeh.2010.00161 PMC2944630

[b15] Elliott C. J. & Benjamin P. R. Interactions of pattern-generating interneurons controlling feeding in *Lymnaea stagnalis*. J. Neurophysiol. 54, 1396–1411 (1985). 4087040 10.1152/jn.1985.54.6.1396

[b16] Gaudry Q. & Kristan W. B. Jr Behavioral choice by presynaptic inhibition of tactile sensory terminals. Nat. Neurosci. 12, 1450–1457 (2009). 19801989 10.1038/nn.2400

[b17] Briggman K. L., Abarbanel H. D. & Kristan W. B. Jr Optical imaging of neuronal populations during decision-making. Science 307, 896–901 (2005). 15705844 10.1126/science.1103736

[b18] Lin S. . Neural correlates of water reward in thirsty *Drosophila*. Nat. Neurosci. 17, 1536–1542 (2014). 25262493 10.1038/nn.3827 PMC4213141

[b19] Mann K., Gordon M. D. & Scott K. A pair of interneurons influences the choice between feeding and locomotion in *Drosophila*. Neuron 79, 754–765 (2013). 23972600 10.1016/j.neuron.2013.06.018 PMC3750742

[b20] Hirayama K. & Gillette R. A neuronal network switch for approach/avoidance toggled by appetitive state. Curr. Biol. 22, 118–123 (2012). 22197246 10.1016/j.cub.2011.10.055 PMC3267890

[b21] Flavell S. W. . Serotonin and the neuropeptide PDF initiate and extend opposing behavioral states in *C. elegans*. Cell 154, 1023–1035 (2013). 23972393 10.1016/j.cell.2013.08.001 PMC3942133

[b22] Nargeot R. & Simmers J. Functional organization and adaptability of a decision-making network in aplysia. Front. Neurosci. 6, 113 (2012). 22855670 10.3389/fnins.2012.00113 PMC3405415

[b23] Gillette R., Huang R. C., Hatcher N. & Moroz L. L. Cost-benefit analysis potential in feeding behavior of a predatory snail by integration of hunger, taste, and pain. Proc. Natl Acad. Sci. USA 97, 3585–3590 (2000). 10737805 10.1073/pnas.97.7.3585 PMC16283

[b24] Krashes M. J. . A neural circuit mechanism integrating motivational state with memory expression in *Drosophila*. Cell 139, 416–427 (2009). 19837040 10.1016/j.cell.2009.08.035 PMC2780032

[b25] Pool A. H. . Four GABAergic interneurons impose feeding restraint in *Drosophila*. Neuron 83, 164–177 (2014). 24991960 10.1016/j.neuron.2014.05.006 PMC4092013

[b26] Inagaki H. K., Panse K. M. & Anderson D. J. Independent, reciprocal neuromodulatory control of sweet and bitter taste sensitivity during starvation in *Drosophila*. Neuron 84, 806–820 (2014). 25451195 10.1016/j.neuron.2014.09.032 PMC4365050

[b27] Sternson S. M., Nicholas Betley J. & Cao Z. F. Neural circuits and motivational processes for hunger. Curr. Opin. Neurobiol. 23, 353–360 (2013). 23648085 10.1016/j.conb.2013.04.006 PMC3948161

[b28] Atasoy D., Betley J. N., Su H. H. & Sternson S. M. Deconstruction of a neural circuit for hunger. Nature 488, 172–177 (2012). 22801496 10.1038/nature11270 PMC3416931

[b29] Krashes M. J. . Rapid, reversible activation of AgRP neurons drives feeding behavior in mice. J. Clin. Invest. 121, 1424–1428 (2011). 21364278 10.1172/JCI46229 PMC3069789

[b30] Kristan W. B. Jr, Calabrese R. L. & Friesen W. O. Neuronal control of leech behavior. Prog. Neurobiol. 76, 279–327 (2005). 16260077 10.1016/j.pneurobio.2005.09.004

[b31] Flood T. F. . A single pair of interneurons commands the *Drosophila* feeding motor program. Nature 499, 83–87 (2013). 23748445 10.1038/nature12208 PMC3727048

[b32] Sippy T., Lapray D., Crochet S. & Petersen C. C. Cell-type-specific sensorimotor processing in striatal projection neurons during goal-directed behavior. Neuron 88, 298–305 (2015). 26439527 10.1016/j.neuron.2015.08.039 PMC4622932

[b33] Longden K. D., Muzzu T., Cook D. J., Schultz S. R. & Krapp H. G. Nutritional state modulates the neural processing of visual motion. Curr. Biol. 24, 890–895 (2014). 24684935 10.1016/j.cub.2014.03.005

[b34] Kemenes G., Elliott C. J. & Benjamin P. R. Chemical and tactile inputs to the *Lymnaea* feeding system: effects on behaviour and neural circuitry. J. Exp. Biol. 122, 113–137 (1986).

[b35] Dawkins M. Behavioural analysis of co-ordinated feeding movements in the gastropod *Lymnaea stagnalis*. J. Comp. Physiol. 92, 255–271 (1974).

[b36] Kemenes G., Hiripi L. & Benjamin P. R. Behavioural and biochemical changes in the feeding system of *Lymnaea* induced by the dopamine and serotonin neurotoxins 6-hydroxydopamine and 5,6-dihydroxytryptamine. Philos. Trans. R. Soc. Lond. B Biol. Sci. 329, 243–255 (1990).

[b37] Vehovszky A., Elliott C. J., Voronezhskaya E. E., Hiripi L. & Elekes K. Octopamine: a new feeding modulator in *Lymnaea*. Philos. Trans. R. Soc. Lond. B Biol. Sci. 353, 1631–1643 (1998).

[b38] Brierley M. J., Staras K. & Benjamin P. R. Behavioral function of glutamatergic interneurons in the feeding system of Lymnaea: plateauing properties and synaptic connections with motor neurons. J. Neurophysiol. 78, 3386–3395 (1997). 9405552 10.1152/jn.1997.78.6.3386

[b39] Evans C. G., Ludwar B. & Cropper E. C. Mechanoafferent neuron with an inexcitable somatic region: consequences for the regulation of spike propagation and afferent transmission. J. Neurophysiol. 97, 3126–3130 (2007). 17267750 10.1152/jn.01341.2006

[b40] Ito E. . Memory trace in feeding neural circuitry underlying conditioned taste aversion in Lymnaea. PLoS ONE 7, e43151 (2012). 22900097 10.1371/journal.pone.0043151 PMC3416747

[b41] Benjamin P. R., Rose R. M., Slade C. T. & Lacy M. G. Morphology of identified neurones in the buccal ganglia of *Lymnaea stagnalis*. J. Exp. Biol. 80, 119–135 (1979).

[b42] Straub V. A., Staras K., Kemenes G. & Benjamin P. R. Endogenous and network properties of Lymnaea feeding central pattern generator interneurons. J. Neurophysiol. 88, 1569–1583 (2002). 12364488 10.1152/jn.2002.88.4.1569

[b43] McCrohan C. R. Initiation of feeding motor output by an identified interneurone in the snail *Lymnaea stagnalis*. J. Exp. Biol. 113, 351–366 (1984).

[b44] McCrohan C. R. Properties of ventral cerebral neurones involved in the feeding system of the snail, *Lymnaea stagnalis*. J. Exp. Biol. 108, 257–272 (1984).

[b45] Staras K., Kemenes G. & Benjamin P. R. Neurophysiological correlates of unconditioned and conditioned feeding behavior in the pond snail *Lymnaea stagnalis*. J. Neurophysiol. 79, 3030–3040 (1998). 9636106 10.1152/jn.1998.79.6.3030

[b46] Yeoman M. S., Vehovszky A., Kemenes G., Elliott C. J. & Benjamin P. R. Novel interneuron having hybrid modulatory-central pattern generator properties in the feeding system of the snail, *Lymnaea stagnalis*. J. Neurophysiol. 73, 112–124 (1995). 7714557 10.1152/jn.1995.73.1.112

[b47] Vavoulis D. V. . Dynamic control of a central pattern generator circuit: a computational model of the snail feeding network. Eur. J. Neurosci. 25, 2805–2818 (2007). 17561845 10.1111/j.1460-9568.2007.05517.x

