# Supplementary material for: A two-neuron system for adaptive goal-directed decision-making in Lymnaea
Source: Nat Commun. 2016 Jun 3;7:11793. doi: 10.1038/ncomms11793 (PMC4895806; doi:10.1038/ncomms11793)
Supplement: Supplementary Information — Supplementary Figures 1-7, Supplementary Methods and Supplementary References [file ncomms11793-s1.pdf]

Supplementary Data

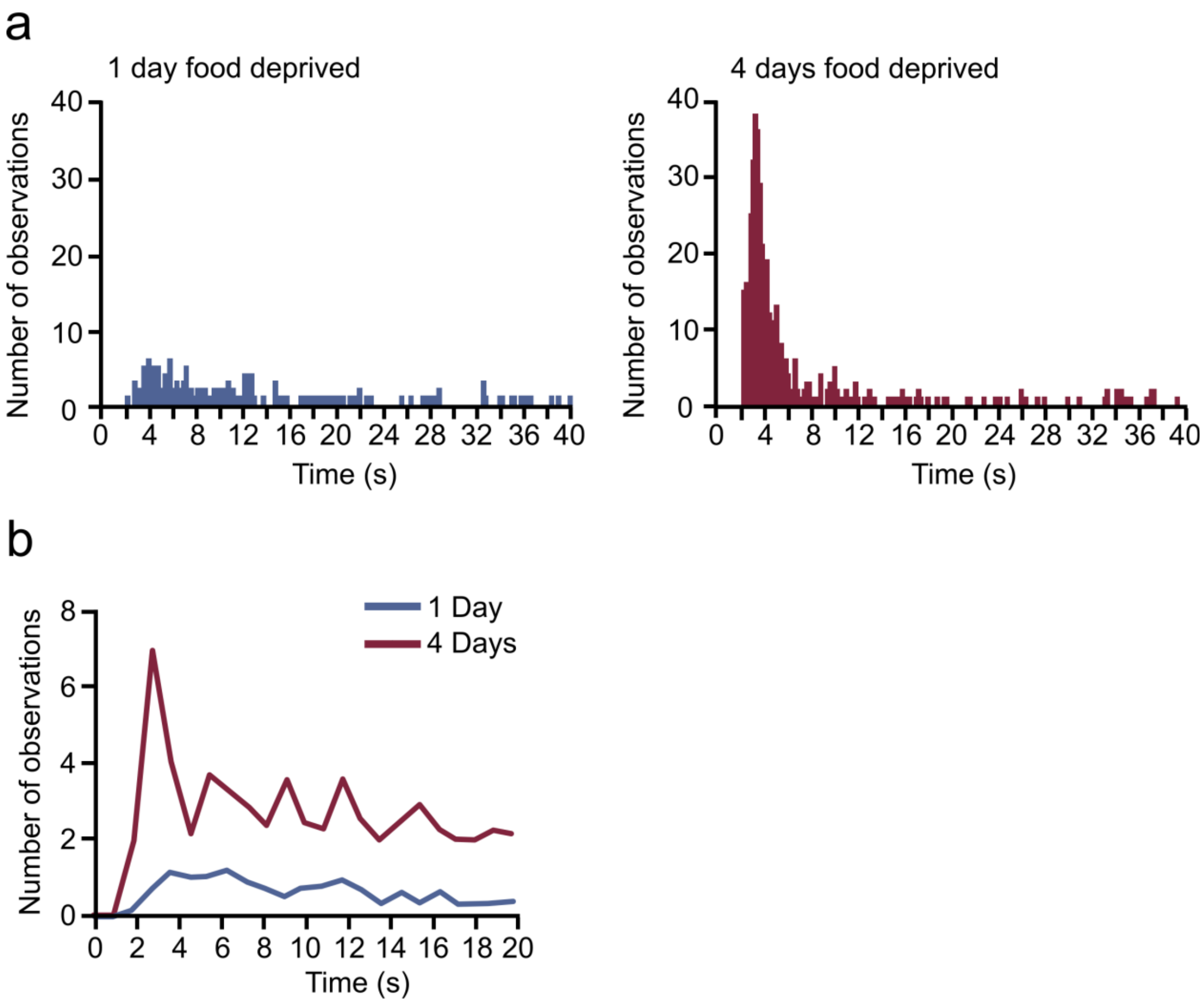

Supplementary Fig. 1. Bite Interval Histogram and Autocorrelogram of appetitive bites.

(**a**) Histograms plotting interval times between onset of an appetitive bite and the next appetitive bite. Data plotted in 0.2 s bins. Histograms are based on all recorded events for *n*=14 (one-day food-deprived) and *n*=14 (four-day food-deprived).

(**b**) Autocorrelogram plot of first 20 appetitive bites from each animal from one-day and four-day food deprived animals. Data plotted in 0.9 s bin widths over a maximal time range of 20 s.

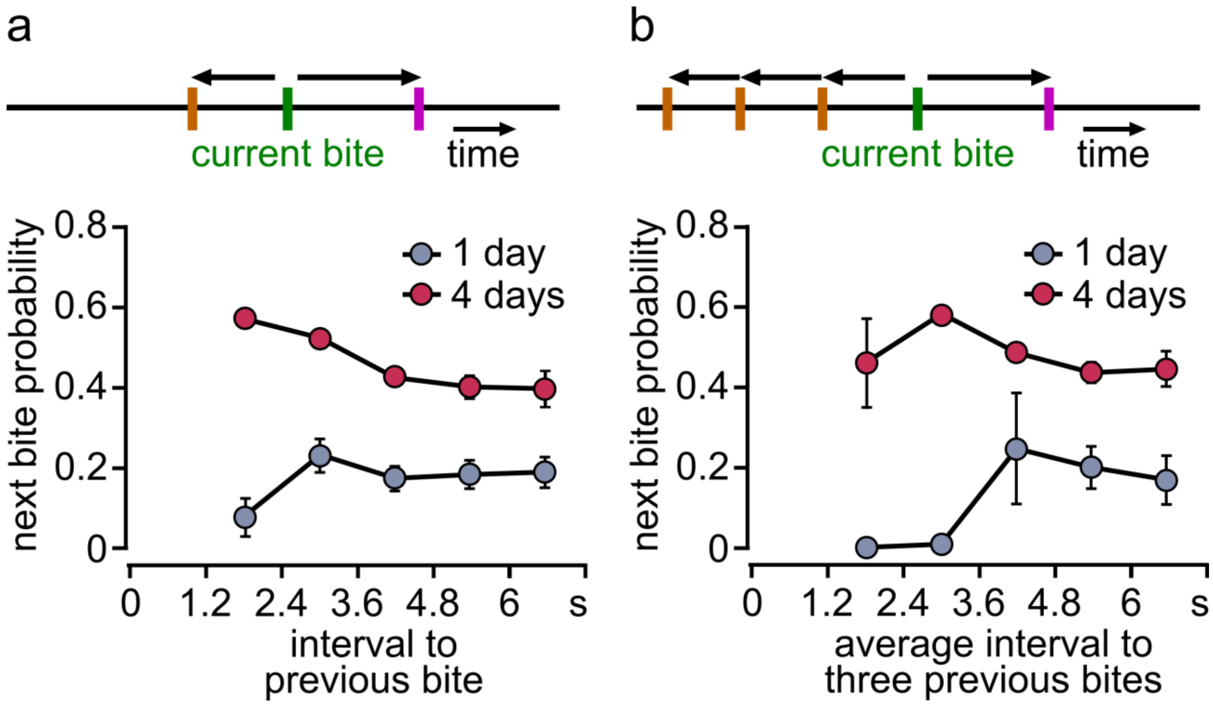

Supplementary Fig. 2. Quantification of bite probability based on previous biting history. (a) For a given bite (green bar, top panel), plot shows mean±SEM probability of next bite event (pink bar, top panel) based on timing interval to previous bite (orange bar, top panel). Bite probability is expressed as the inverse of next bite interval/lowest bite interval (1.99 s). Data plotted in 1.2 s bins. **(b)** as in (a) but using bite history based on average of three previous bite intervals. Plots are based on all recorded events for *n*=14 (one-day food-deprived) and *n*=14 (four-day food-deprived).

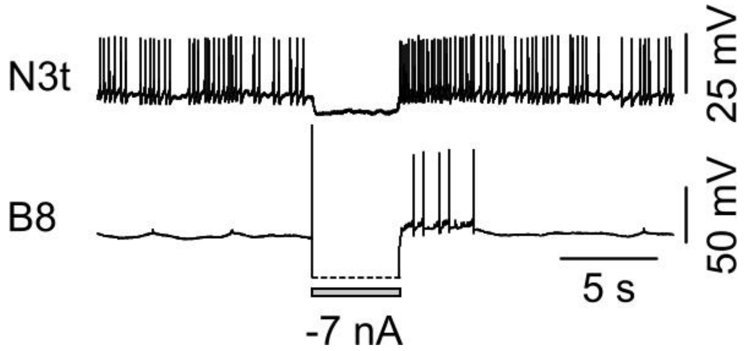

Supplementary Fig. 3. N3t can be silenced by the hyperpolarization of an electrotonically coupled motoneuron. Injecting hyperpolarising current into a B8 motoneuron causes a similar yet attenuated hyperpolarisation of N3t which prevents the cell from firing tonically (*n*=4). Grey bar represents the duration of hyperpolarising current injection into B8.

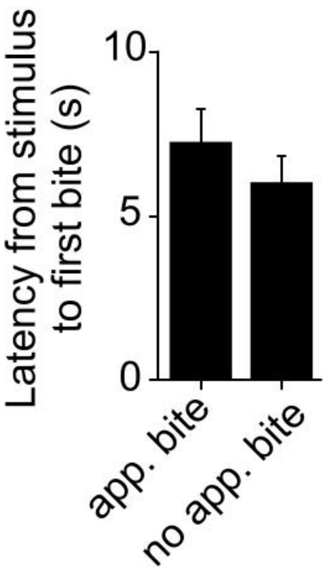

Supplementary Fig. 4. A chemical food stimulus has the same delayed effect whether applied during or in the absence of an appetitive bite. In all animals tested in Fig. 4a,b, the chemical stimulus applied during an appetitive bite initiated a bout of feeding cycles, indicating that it is sufficient to elicit feeding behavior. However, the long latency onset provides evidence that it is not the most important sensory modality involved in the stimulus-present decision during the appetitive bite behavior. Application of the same chemical stimulus to the mouth region of the animal in the absence of an appetitive bite also initiated a bout of feeding cycles characterized by a delay until onset of the first bite from the application of the chemical stimulus. The latency of the first bite when lettuce juice was applied to the mouth of animals (n=15) during an appetitive bite and when it was applied in the absence of an appetitive bite was not significantly different. Paired t-test, $P>0.05$ (n.s.).

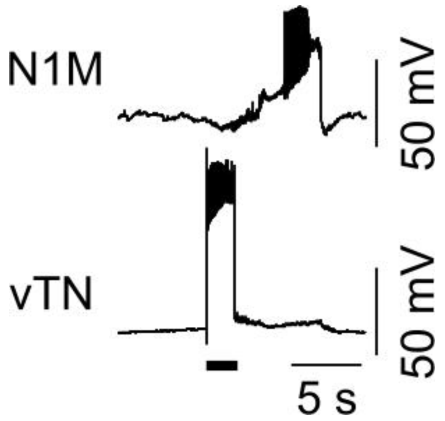

Supplementary Fig. 5. Activation of vTN results in the generation of a full fictive feeding cycle by N1M. A burst of spikes evoked by the injection of depolarizing current into vTN (black bar) triggers a plateau potential in N1M that leads to the generation of a fictive feeding cycle. This is an example from 11 independent experiments showing the same result.

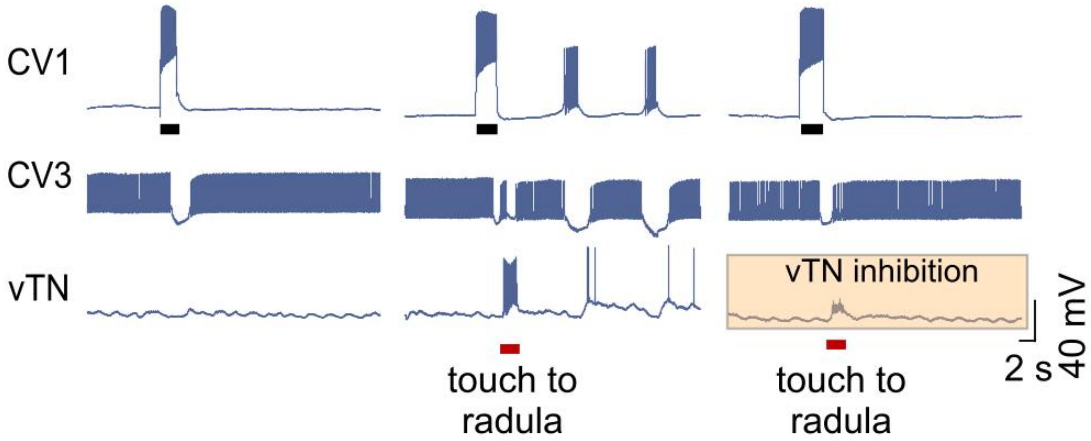

Supplementary Fig. 6. vTN activity is necessary for stimulus present decision. Representative traces of neurons CV1, CV3 and vTN from an experiment testing the importance of vTN for the stimulus present decision. (Left traces) A burst of spikes in CV1 in the absence of a tactile stimulus (touch to the radula) is not sufficient to trigger associated fictive feeding bites. (Middle traces) A tactile stimulus was applied to the radula in a radula-CNS preparation in the swallow phase of a fictive appetitive bite triggering a burst of somatic spikes in vTN and initiating associated cycles as seen by the rhythmic activity in CV1 itself and the lip closer motoneuron CV3[1]. (Right traces) Application of the tactile stimulus to the radula whilst vTN was hyperpolarised (orange box) to prevent somatic spiking (right trace) was insufficient to initiate associated cycles. Black bars represent the duration of depolarising current injection into CV1, red bars represent duration of tactile stimulation to the radula.

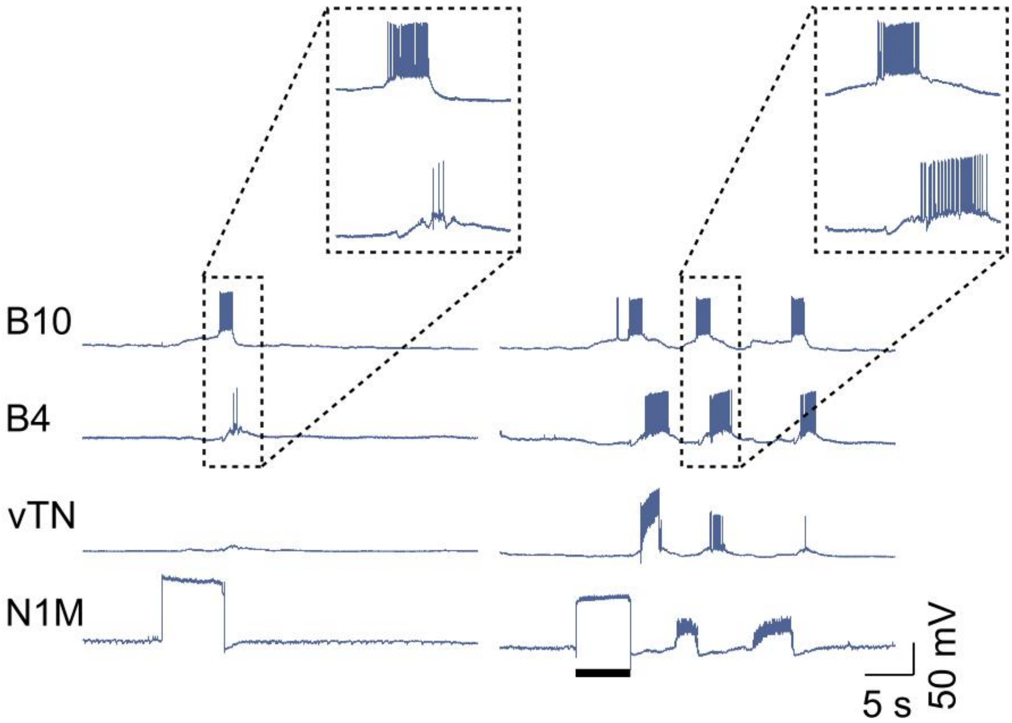

Supplementary Fig. 7. Stimulus present decisions do not alter the motoneuronal output of the major rasp phase motoneuron B10. The two (left and right) sets of four co-recorded traces show representative examples of B10, B4, vTN and N1M activity with a stimulus absent (left) and a fictive stimulus present (right) trial from a preparation from a one-day food-deprived animal. Boxed areas show an expanded trace of B10 and B4 activity in the second cycle and indicate the time window analysed in the experiment. The second cycle was analysed when vTN was activated after B10 was active in the first cycle. There was no significant difference in B10 activity between the fictive stimulus present and absent trials. Paired t-test, P>0.05 (*n*=5).

Supplementary Methods

Testing vTN's role in the stimulus-present decision using a physiologically relevant stimulus

To test the importance of vTN for the stimulus present decision, the semi-intact radula-CNS preparation was used (see Methods). However, this preparation did not allow for co-recording of vTN with N1M due to the two neurons being located on opposite surfaces of the buccal ganglia and the radula-CNS preparation not being suitable for a twisted buccal preparation. Instead, a cerebral to buccal command-like interneuron (CV1[2]) was used to initiate cycles. Activation of a single CV1 interneuron until the onset of the N2 phase, similar to N1M activation, was sufficient to initiate a fictive feeding cycle. To ensure that CV1 was suitable to use in these experiments we compared the percentage of fictive appetitive bites with an associated bite in one-day food-deprived preparations with those observed behaviorally in one-day food-deprived animals and found no significant difference (CV1 electrophysiology, 13±6% (*n*=13); Behavioral observation, 15±4% (*n*=14); Unpaired t-test, P>0.05).

Supplementary References

1. McCrohan CR. Properties of ventral cerebral neurones involved in the feeding system of the snail, *Lymnaea stagnalis*. *J Exp Biol* **108**, 257-272 (1984).

2. McCrohan CR. Initiation of feeding motor output by an identified interneurone in the snail *Lymnaea stagnalis*. *J Exp Biol* **113**, 351-366 (1984).
